# Gabapentinoids and Risk of Hip Fracture

**DOI:** 10.1001/jamanetworkopen.2024.44488

**Published:** 2024-11-13

**Authors:** Miriam T. Y. Leung, Justin P. Turner, Clara Marquina, Jenni Ilomäki, Tim Tran, Katsiaryna Bykov, J. Simon Bell

**Affiliations:** 1 Centre for Medicine Use and Safety, Faculty of Pharmacy and Pharmaceutical Sciences, Monash University, Melbourne, Australia; 2 Faculty of Pharmacy, University of Montreal, Quebec City, Quebec, Canada; 3 Centre de Recherche, Institut Universitaire de Gériatrie de Montréal, Montréal, Québec, Canada; 4 Faculty of Pharmacy, Laval University, Quebec City, Quebec, Canada; 5 Department of Epidemiology and Preventive Medicine, Monash University, Melbourne, Australia; 6 Pharmacy Department, Austin Health, Melbourne, Australia; 7 Division of Pharmacoepidemiology and Pharmacoeconomics, Department of Medicine, Brigham and Women’s Hospital and Harvard Medical School, Boston, Massachusetts; 8 Faculty of Health Sciences, University of Eastern Finland, Kuopio, Finland

## Abstract

**Importance:**

The increased use of gabapentinoids has been most pronounced in older people who are also susceptible to hip fractures.

**Objective:**

To investigate the overall association between gabapentinoids and the risk of hip fractures and the stratified association across age groups, frailty status, and history of chronic kidney disease.

**Design, Setting, and Participants:**

This was a case-case-time-control study in patients hospitalized for hip fracture in Victoria, Australia, between March 1, 2013, and June 30, 2018, with at least 1 prescription for a gabapentinoid before fracture. Conditional logistic regression was used to estimate the odds ratio (OR) and 95% CI for gabapentinoid dispensing in the index (1-60 days prefracture) compared with the reference (121-180 days prefracture) period. To adjust for the underlying time trend in gabapentinoid use, each index case was matched with up to 5 controls, selected from future cases of the same age and sex. Subgroup analyses were conducted in subgroups with or without chronic kidney disease (CKD), frailty scores less than 5, and frailty scores 5 and above. Frailty was computed using the Hospital Frailty Risk Score (HFRS). Data were analyzed from November 2023 to April 2024.

**Exposure:**

Gabapentinoids (pregabalin or gabapentin).

**Main Outcome and Measure:**

Hip fracture.

**Results:**

Of 28 293 patients hospitalized for hip fractures, 2946 (1752 [59.5%] aged ≥80 years; 2099 [71.2%] female) were dispensed a gabapentinoid before hip fracture. Gabapentinoid dispensing was associated with increased odds of hip fractures (OR, 1.96; 95% CI, 1.66-2.32). After adjusting for the exposure-time trend and concomitant use of other central nervous system medications, the odds of hip fractures remained elevated (OR, 1.30; 95% CI, 1.07-1.57). The association between gabapentinoid dispensing and hip fracture was higher in patients with HFRS 5 and above (OR, 1.75; 95% CI, 1.31-2.33) and CKD (OR, 2.41; 95% CI, 1.65-3.52).

**Conclusions and relevance:**

In this case-case-time-control study of Australian residents hospitalized for hip fracture, gabapentinoid use was associated with an increased risk of hip fractures, especially in patients who were frail or had chronic kidney disease. In addition to the known risk associated with kidney impairment, frailty status may be an important risk factor when considering use of gabapentinoids.

## Key Points

**Question:**

Is the use of gabapentinoids associated with risk of hip fracture, and does the risk differ across age, frailty, and kidney function?

**Findings:**

In this case-case-time-control study including 28 293 patients hospitalized for hip fractures in Australia, gabapentinoid use was associated with an increased risk of hip fracture, especially in patients who were frail and had chronic kidney disease.

**Meaning:**

These findings suggest that in addition to the known risk associated with kidney impairment, gabapentinoids should be used with caution among patients at risk of hip fractures, especially those who are frail.

## Teaser

This case-case-time-control study evaluates the association between gabapentinoids and the risk of hip fractures among older adults in Australia.

## Introduction

The exponential increase in gabapentinoid use has been particularly pronounced among older adults, especially after the expansion of the indication to include various painful syndromes (eg, neuropathic pain and fibromyalgia).^1,2,3,4^ By 2018, gabapentin was the sixth most dispensed medication by volume in the US,^5^ with utilization continuing to increase until 2021.^2^ A corresponding 8-fold increase in prescriptions for gabapentinoids in Australia was observed from 2012 to 2018, with 1 in 7 Australians aged 80 and older prescribed a gabapentinoid across this period.^4^ Pregabalin remains within the 10 most subsidized medications by volume in Australia in 2023.^6^

Gabapentinoids have been marketed as a safer alternative to opioids for the treatment of neuropathic pain.^7,8^ This may be partly responsible for decreasing opioid and increasing gabapentin use in US veterans between 2015 and 2019.^9^ However, more evidence is needed regarding gabapentinoids’ adverse drug event (ADE) profile in older people. Gabapentinoids are actively transported across the blood-brain barrier and inhibit neurotransmitter release via multiple pathways.^7,10^ This explains why gabapentinoids have efficacy for various central nervous system (CNS) disorders, including seizures and neuropathic pain.^7,10^ It is also why gabapentinoids have CNS ADEs including somnolence, dizziness, gait disturbance, and balance disorder.^11,12^ These adverse events may increase the risk of falls and fractures in older people. Hip fractures are associated with the highest costs to individuals and health systems among all fragility fractures.^13,14^ One in 25 people aged 80 years or older experience a hip fracture each year,^15^ with 1 in 4 dying within 12 months.^14^ However, there is limited evidence regarding the possible association between gabapentinoids and hip fractures.

Selection of an appropriate medication can be informed by knowledge about patient factors that are associated with an increased risk of ADEs.^16^ Frailty and kidney impairment are 2 important considerations when prescribing for older people and may occur together or separately.^17,18,19^ Frailty is increasingly recognized as a useful clinical parameter for risk stratification.^20^ Frailty has also been associated with an increased risk of hip fractures.^21,22,23,24^ However, no studies have investigated whether frailer patients dispensed gabapentinoids are at greater risk of hip fractures than less frail patients. Similarly, gabapentinoids are predominately excreted through the kidneys and have a prolonged elimination half-life in patients with kidney impairment. No studies have investigated whether patients with chronic kidney disease (CKD) are at increased risk of hip fracture associated with gabapentinoids. The objective of our study was to investigate the overall association between gabapentinoids and the risk of hip fractures and the stratified association across age groups, frailty status, and history of CKD.

## Methods

The study was approved by Australian Institute of Health and Welfare (AIHW) ethics committee and Monash University human research ethics committee. Informed consent was waived by all data custodians as data were deidentified. The study follows the Strengthening the Reporting of Observational Studies in Epidemiology (STROBE) reporting guideline.^25^

### Data Sources

We extracted data from 4 linked administrative datasets: the Victorian Admitted Episodes Dataset (VAED), the Victorian Emergency Minimum Dataset (VEMD), Pharmaceutical Benefits Scheme (PBS), and the National Death Index (NDI). The VAED contains information on all public and private hospitalizations in Victoria, the second most populous state in Australia. Information available includes patient demographic and diagnostic data. The VEMD contains information on all emergency visits to the hospitals. The PBS dataset contains information on all medications subsidized by the Australian government and dispensed at community pharmacies, outpatient clinics or at hospital discharge for Australian residents. The NDI dataset contains all deaths registered across Australia. Data were available from July 1, 2006, to June 30, 2018, for all datasets. Details on the datasets have been published previously.^14,26,27^

### Study Population

Our study included all patients aged 50 years or older admitted for first hip fracture (*International Statistical Classification of Diseases and Related Health Problems, Tenth Revision, Australian Modification [ICD-10-AM]* codes S72.0-S72.2) between March 1, 2013, and June 30, 2018, with at least 1 dispensing of gabapentinoids (*World Health Organization Anatomical Therapeutic Chemical Classification, Australian Modification* [*ATC*] codes N02BG and N03AX) ever recorded before the fracture. First hip fracture was defined as an absence of hip fracture in the 5 years before their admission and the date of admission was defined as the index date. The cohort of patients with first hip fracture has been described previously.^14^ We also excluded anyone whose exposure assessment period started before the pregabalin was first PBS-subsidized on March 1, 2013.^4^ Furthermore, we excluded patients hospitalized for more than 50% of their exposure assessment period because PBS data do not capture inpatient dispensings.

### Study Design

**Figure 1.**
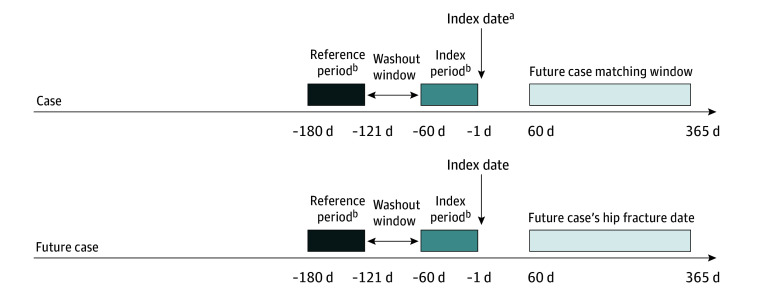
Schematic Representation of Case-Case-Time-Control Study Design ^a^The exclusion window was 5 years to 1 day before the index date. The comorbidities assessment window was 5 years before the index date to the baseline assessment window (day 0). The frailty assessment window was 2 years to 1 day before the index date. ^b^Index and reference periods were the exposure assessment periods for gabapentinoids and time-varying covariates (ie antidepressants, antipsychotics, benzodiazepines, and opioids).

The case-case-time-control analysis consisted of 2 case-crossover analyses, (1) the case case-crossover analysis and (2) the future-case-control case-crossover analysis (Figure 1).^28,29,30^ Case-crossover is a self-controlled design that compares exposure to the drug of interest within the same patient across different time periods (index period immediately before event vs a reference period before the index period), thereby controlling for confounders that are stable across the time periods by design.^30^ Since only patients who have exposure to the drug of interest (gabapentinoids) contribute to case-crossover analyses, we restricted the study population to cases (patients with hip fracture) who had prior exposure to gabapentinoids. Self-controlled study designs are advantageous in the presence of unmeasured patient characteristics (eg, bone health, muscle strength, nutritional status, and mobility status) that would confound conventional study designs, such as cohort and case-control.^31,32^

In the case case-crossover analysis, the odds of dispensing gabapentinoids during the index period (1 day to 60 days preceding the index date) were compared with the odds of dispensing in the reference period (121 to 180 days preceding the index date). Medication exposure was ascertained from dispensings recorded in the PBS dataset. A 60-day period duration was chosen as each dispensing on the PBS is typically for a 1-month supply. This was in accordance with a previous study on utilization of gabapentinoids in Australia.^33^ A 60-day gap (days 61 to 120 before the index date) was applied between index period and reference period as a washout period to minimize any carryover effect from leftover medications in the reference period. Dispensing was assessed only during the index and reference, but not the washout period. The role of the washout period was to space apart the index and reference periods to avoid contamination from any leftover medications carrying over from reference period to index period.

Case-crossover analysis may produce biased results in the presence of increasing time trends in population drug use or when the drug of interest is used chronically.^30,34^ Since previous research suggests increasing use of gabapentinoids during our study period and around one-third of Australian patients are persistent gabapentinoid users,^33^ we implemented a case-case-time-control study design that uses future cases as controls.^29^ This design has been shown to control for time trends in exposure and persistent user bias in case-crossover analyses.^35^

The future-case-control case-crossover analysis considers all cases before their event (future cases) to be part of the at-risk population eligible as controls, approximating case-only incidence density sampling.^36^ The use of future cases as present controls also minimizes bias from different baseline risks across cases and controls, as the controls were sampled from soon-to-be cases with more comparable risks than external controls.^28,29,30^ Risk-set sampling was used to match up to 5 future cases for each case as controls, based on age (±5 years for ages <85 or ≥85) and sex. To ensure comparable risks, controls were further restricted to patients with hip fractures occurring 60 to 365 days after the matched case index dates. Cases with index dates after May 1, 2018, were precluded from our matched analysis due to unavailability of future cases for matching.

### Subgroup Analyses

Patient frailty status was assessed using the Hospital Frailty Risk Score (HFRS). The HFRS is a validated deficit accumulation frailty score computed as a weighted sum of 109 diagnoses recorded within 2 years before the index date.^37^ Variation in susceptibility to gabapentinoids by frailty status was assessed by conducting subgroup analyses in those with low frailty risk (HFRS<5) and high frailty risk (HFRS≥5).^26,37^ To assess the modifying effects of age and CKD, analyses were conducted separately in subgroups of patients aged younger than 80 years or 80 years or older, as well as patients with or without CKD. CKD was defined as a diagnosis for CKD or kidney failure (according to AIHW) within 5 years before the index date (eTable 1 in Supplement 1).^38^

### Time-Varying Covariates

The use of other CNS-active fall-risk-increasing medications could confound the association between gabapentinoids and hip fracture. Therefore, we adjusted for concomitant dispensing of antidepressants (ATC N06A), antipsychotics (ATC N05A), benzodiazepines (ATC N03AE, N05BA, and N05CD) and opioids (ATC N02A).^39,40,41^

### Comorbidities Ascertainment

Comorbidities were ascertained from the diagnoses recorded in the VAED and VEMD dataset. Comorbidities were defined as any diagnosis recorded in the 2 datasets within 5 years before the index date (eTable 1 in Supplement 1).

### Statistical Analysis

Baseline characteristics of cases and future-case-controls were characterized to assess the comparability of cases and controls. The comparability of baseline characteristics was assessed using standardized mean difference (SMD). Characteristics with SMDs less than 0.1 were considered similar. The odds ratio (OR) and 95% CI between gabapentinoid dispensing and hip fractures were estimated using a logistic regression conditioned on an individual. The time-varying covariates were added to the model as covariates. The case-case-time-control OR was estimated using the interaction term between exposure status and an indicator for cases vs controls. To assess the robustness of the results, additional sensitivity analyses were conducted by varying the lengths of time periods. The lengths of exposure assessment periods (index and reference periods) were altered from 60 days to 30 and 90 days, while the length of washout window was altered from 60 days to 30, 90, 120, 150, and 180 days. A statistically significant difference between subgroups was defined as nonoverlapping 95% CIs. All analyses were performed using SAS version 9.4 (SAS Institute) and R version 4.0.0 (R Project for Statistical Computing). Data were analyzed from November 2023 to April 2024.

## Results

### Characteristics of Study Population

**Figure 2.**
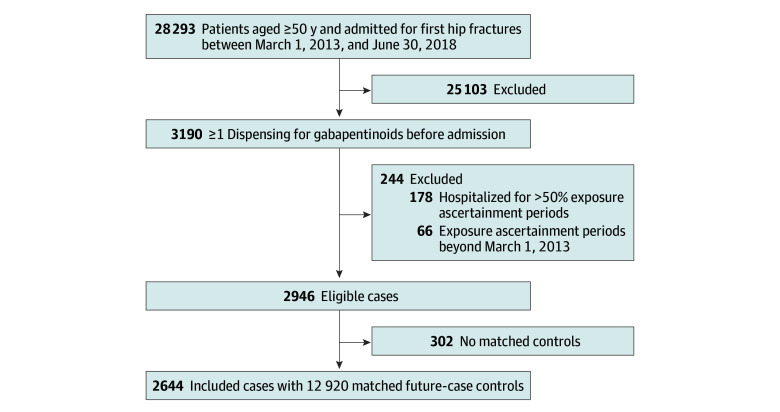
Study Flow Diagram

Overall, 28 293 patients (18 759 [66%] aged ≥80 years; 19 357 [69%] female) were hospitalized for first hip fractures in Victoria, Australia, from March 1, 2013, to June 30, 2018. Of these, 3190 patients were dispensed gabapentinoids before being admitted. Most patients used pregabalin (2995 patients [93.9%]). After excluding patients with incomplete data or who were hospitalized for more than 50% of the exposure ascertainment period, 2946 patients (1752 [59.5%] aged ≥80 years; 2099 [71.2%] female) were eligible for inclusion in the main analysis. In the analysis, 2644 patients were matched with at least 1 future case as a control (Figure 2). Most of the cases were female (1887 patients [71.4%]). More than half were 80 years or older (1579 patients [59.7%]). The cases and future cases were comparable in terms of age, sex, and comorbidities (Table 1).

**Table 1.** Characteristics of All Eligible Patients, Patients Included, and Matched Future-Case-Controls

Characteristic	Patients, No. (%)			Standardized mean difference
	All eligible cases (N = 2946)	Cases included (n = 2644)	Matched controls (n = 12 920)	
Age, y				0.039
50-59	128 (4.3)	109 (4.1)	441 (3.4)	
60-69	318 (10.8)	281 (10.6)	1356 (10.5)	
70-79	748 (25.4)	675 (25.5)	3290 (25.5)	
80-84	605 (20.5)	536 (20.3)	2667 (20.6)	
≥85	1147 (38.9)	1043 (39.4)	5166 (40.0)	
Sex				0.020
Male	847 (28.8)	757 (28.6)	3583 (27.7)	
Female	2099 (71.2)	1887 (71.4)	9337 (72.3)	
HFRS				0.002
<5	1634 (55.5)	1456 (55.1)	7128 (55.2)	
≥5	1312 (44.5)	1188 (44.9)	5792 (44.8)	
Comorbidities				
Anxiety	234 (7.9)	210 (7.9)	1029 (8.0)	0.001
Arthritis	609 (20.7)	546 (20.7)	2755 (21.3)	0.017
Alcohol use disorder	107 (3.6)	99 (3.7)	462 (3.6)	0.009
Cancer	578 (19.6)	514 (19.4)	2616 (20.2)	0.020
Cardiovascular disease	1887 (64.1)	1687 (63.8)	8464 (65.5)	0.036
Cerebrovascular disease	299 (10.1)	273 (10.3)	1366 (10.6)	0.008
Chronic kidney disease	915 (31.1)	811 (30.7)	4301 (33.3)	0.056
COPD/asthma	416 (14.1)	376 (14.2)	1826 (14.1)	0.003
Delirium	471 (16.0)	424 (16.0)	2074 (16.1)	<0.001
Dementia	129 (4.4)	116 (4.4)	630 (4.9)	0.023
Depression or other affective disorders	192 (6.5)	175 (6.6)	830 (6.4)	0.008
Diabetes	773 (26.2)	704 (26.6)	3484 (27.0)	0.008
Epilepsy/seizures	88 (3.0)	77 (2.9)	351 (2.7)	0.012
Fall	988 (33.5)	881 (33.3)	4455 (34.5)	0.025
Gastroesophageal reflux disease	292 (9.9)	262 (9.9)	1235 (9.6)	0.012
Osteoporosis	865 (29.4)	776 (29.3)	3820 (29.6)	0.005
Peptic ulcer disease	106 (3.6)	100 (3.8)	468 (3.6)	0.008
Previous vertebral fracture	178 (6.0)	155 (5.9)	954 (7.4)	0.061
Visual disturbances and blindness	100 (3.4)	89 (3.4)	413 (3.2)	0.010
Time-varying exposure				
Gabapentinoids				
Index period	1313 (44.6)	1208 (45.7)	5927 (45.9)	0.004
Index period only	425 (14.4)	402 (15.2)	1343 (10.4)	0.144
Reference period	1107 (37.6)	1011 (38.2)	5499 (42.6)	0.088
Reference period only	219 (7.4)	205 (7.8)	915 (7.1)	0.026
Antidepressants				
Index period	1287 (43.7)	1163 (44.0)	4811 (37.2)	0.138
Reference period	1191 (40.4)	1077 (40.7)	4672 (36.2)	0.094
Antipsychotics				
Index period	180 (6.1)	164 (6.2)	582 (4.5)	0.075
Reference period	147 (5.0)	135 (5.1)	519 (4.0)	0.052
Benzodiazepines				
Index period	667 (22.6)	609 (23.0)	2647 (20.5)	0.062
Reference period	648 (22.0)	584 (22.1)	2667 (20.6)	0.035
Opioids				
Index period	1491 (50.6)	1352 (51.1)	5374 (41.6)	0.192
Reference period	1329 (45.1)	1208 (45.7)	5195 (40.2)	0.111

Abbreviation: COPD, chronic obstructive pulmonary disease; HFRS, Hospital Frailty Risk Score.

### Odds of Hip Fractures

Among the 2644 matched cases, 607 had crossover in exposure across the index and reference periods (ie, discordant exposure pattern), with 402 dispensed gabapentinoids in the index period only (60 days to 1 day before index admission) and 205 dispensed in the reference period only (180 to 121 days before index admission). Gabapentinoid dispensing was associated with increased odds of hip fracture (OR, 1.96; 95% CI, 1.66-2.32). For the controls, 1343 were dispensed gabapentinoids in the index period only and 915 in the reference period only, estimating an underlying time-trend of more gabapentinoid dispensing during the index period (OR, 1.47; 95% CI, 1.35-1.60). The time-trend adjusted case-case-time-control OR was 1.34 (95% CI, 1.11-1.61), and further adjustment for time-varying dispensing of other CNS-active medications resulted in an OR of 1.30 (95% CI, 1.07-1.57) (Table 2).

**Table 2.** Results of Case-Case-Time-Control Analyses for Main and Subgroup Analyses

Characteristic	Patients, No.		Odds ratio (95% CI)
	Exposed during index period only	Exposed during reference period only	
Overall			
Case crossover	402	205	1.96 (1.66-2.32)
Control crossover	1343	915	1.47 (1.35-1.60)
Case-case-time-control	NA	NA	1.34 (1.11-1.61)
Adjusted case-case-time-control^a^	NA	NA	1.30 (1.07-1.57)
Age			
<80 y			
Case crossover	167	84	1.99 (1.53-2.58)
Control crossover	542	368	1.47 (1.29-1.68)
Case-case-time-control	NA	NA	1.35 (1.00-1.81)
Adjusted case-case-time-control^a^	NA	NA	1.34 (1.00-1.80)
≥80 y			
Case crossover	235	121	1.94 (1.56-2.42)
Control crossover	801	547	1.46 (1.31-1.63)
Case-case-time-control	NA	NA	1.33 (1.04-1.69)
Adjusted case-case-time-control^a^	NA	NA	1.25 (0.98-1.61)
Frailty			
HFRS: <5			
Case crossover	206	115	1.79 (1.43-2.25)
Control crossover	641	464	1.38 (1.23-1.56)
Case-case-time-control	NA	NA	1.29 (1.00-1.68)
Adjusted case-case-time-control^a^	NA	NA	1.26 (0.98-1.64)
HFRS: ≥5			
Case crossover	192	89	2.16 (1.68-2.77)
Control crossover	492	415	1.19 (1.04-1.35)
Case-case-time-control	NA	NA	1.82 (1.37-2.42)
Adjusted case-case-time-control^a^	NA	NA	1.75 (1.31-2.33)
Without CKD			
Case crossover	283	157	1.80 (1.48-2.19)
Control crossover	932	591	1.58 (1.42-1.75)
Case-case-time-control	NA	NA	1.14 (0.92-1.43)
Adjusted case-case-time-control^a^	NA	NA	1.13 (0.90-1.41)
With CKD			
Case crossover	116	47	2.47 (1.76-3.46)
Control crossover	283	294	0.96 (0.82-1.13)
Case-case-time-control	NA	NA	2.56 (1.76-3.73)
Adjusted case-case-time-control^a^	NA	NA	2.41 (1.65-3.52)

Abbreviations: CKD, chronic kidney disease; HFRS, Hospital Frailty Risk Score; NA, not applicable.

^a^ Adjusted for time-varying exposure to antidepressants, antipsychotics, benzodiazepines, and opioids.

### Subgroup Analyses

Among those with CKD (1036 patients) or high frailty risk (1519 patients), 780 had both CKD and high frailty risk. Higher odds of hip fracture were found among patients with high frailty risk (OR, 1.75; 95% CI, 1.31-2.33) than among patients with low frailty risk (OR, 1.26; 95% CI, 0.98-1.64). Subgroup analysis for patients with and without chronic kidney diseases demonstrated that the risk of hip fractures associated with gabapentinoid dispensing was significantly higher among patients with CKD (OR, 2.41; 95% CI, 1.65-3.52) than those without CKD (OR, 1.13; 95% CI, 0.90-1.41). However, when stratified by age group, the higher risk of hip fractures remained similar across different age groups without reaching statistical significance due to smaller sample sizes (50-79 years old: OR, 1.34; 95% CI, 1.00-1.80; ≥80 years old: OR, 1.25; 95% CI, 0.98-1.61) (Table 2; eTable 2 in Supplement 1).

### Sensitivity Analyses

The characteristics of cases and controls for each sensitivity analysis were similarly comparable (eTables 3-6 in Supplement 1). Results remained similar across sensitivity analyses, from the most restrictive analysis that used the shortest exposure assessment periods and washout period of 30 days (adjusted case-case-time-control OR, 1.40; 95% CI, 1.15-1.71) to the sensitivity analysis of the longest exposure assessment periods of 90 days and washout period of 180 days (adjusted case-case-time-control OR, 1.45; 95% CI, 1.23-1.70) (eTable 7 in Supplement 1).

## Discussion

In this population-based self-controlled case-case-time-control study, we found gabapentinoid dispensing was associated with higher odds of hip fracture. The association was similar across age groups but higher in those who were frailer or had CKD. Our study suggests that frailty and CKD may be important factors for clinicians to consider when prescribing gabapentinoids.

Our results highlight that patients had 30% increased odds of hip fractures within 60 days of gabapentinoid dispensing. In comparison, previous case-crossover studies on hip fractures demonstrated 124% increased odds with antidepressant use,^42^ 47% increased odds with antipsychotic use,^43^ 55% to 75% increased odds with benzodiazepine use,^44^ and 62% increased odds with opioid use.^43^ However, these case-crossover studies did not include any control case-crossover analyses to account for possible bias arising from temporal trends in medication dispensing.^42,43,44^ A systematic review in 2020^45^ reported association with hip fractures for psychotropic medications ranging from a pooled OR of 1.33 in sedatives to 2.36 in antiparkinsonian drugs. Recent systematic reviews reported similar increased odds of hip fractures in benzodiazepine users with a pooled OR of 1.32 across case-control studies,^46^ and in tramadol users with a pooled hazard ratio of 1.32 across cohort studies.^47^ Our results suggest that gabapentinoids may be associated with a similar risk of hip fracture as other fall-risk-increasing medications.^48^ In addition to previous studies on antiseizure medications and fragility fractures,^49,50,51,52^ our study quantifies the specific risk associated with gabapentinoids. Such specificity is useful as fragility fractures vary widely in terms of treatment and prognosis, wherein hip fractures entail the largest morbidity and mortality burden.^13,53^ In light of increasing gabapentinoid prescribing, our results have important implications for clinicians and policy makers.

To our knowledge, this is the first study to highlight that gabapentinoids are associated with higher odds of hip fracture in patients who are frail. This substantiates existing evidence that frail people are more susceptible to adverse drug events.^17,54^ Previous studies have shown that frailty score calculators could be successfully integrated into electronic health records to aid clinicians in decision-making at point-of-care.^55,56,57^ Our findings suggest a potential utility of frailty risk scores as a risk stratification tool and the importance of assessing risks of patients individually before prescribing gabapentinoids.

Our study also reports higher odds of hip fractures in patients with CKD who were dispensed gabapentinoids. In contrast, clinical trials on gabapentinoids have reported no increased risk of hip fractures.^11,12,58^ A recent systematic review suggested higher risks of adverse drug events (eg, lethargy, drowsiness, dizziness, fall, and fractures) among patients with CKD.^59^ However, specific studies on the association between hip fractures and gabapentinoids in CKD are scarce, with mainly 1 large observational study finding an increased risk of hip fracture in patients using high-dose gabapentinoids while receiving hemodialysis.^59,60,61^ American Geriatrics Society Beers Criteria recommend dosage adjustment in patients with moderate and/or severe kidney impairment who are prescribed gabapentinoids. This is because gabapentinoids could accumulate in patients with kidney impairment and cause CNS ADEs.^39^ The higher risk we observed may be due to insufficient dosage adjustment or residual risk remaining after dose adjustment of gabapentinoids for patients with CKD; however, we were unable to investigate the modifying effect of doses in our study.^62^ Further studies examining the risk of hip fractures associated with different dosages of gabapentinoids among patients with different degrees of kidney impairment or dialysis status are needed.

### Strengths and Limitations

This study has several strengths. Our study used a self-controlled study design that inherently eliminates confounding from variations across individuals (eg, baseline frailty and kidney function). The unidirectional sampling of the reference period for exposure also avoided bias arising from deprescribing of gabapentinoids after hip fractures. Additionally, our case-case-time-control design used future cases to account for temporal trend and persistent user bias, 2 common sources of bias in case-crossover analyses.^29,34^ This was a population-based study using administrative databases, thus minimizing the risk of selection bias. The similar results from our various sensitivity analyses also assures the robustness of our results.

Our study also has several limitations. We analyzed medication dispensing and could not ascertain whether dispensed medications were taken by the patients, nor the dosages taken. Case-crossover is better suited to assess acute drug effects than long-term or cumulative effects. While most adverse drug effects of gabapentinoids related to falls (eg, dizziness and somnolence) were reported within 1 to 2 weeks of gabapentinoid initiation,^11^ gabapentinoids may also have long-term adverse effects on bone health resulting from interference with calcium homeostasis.^63^ Our results may, therefore, underestimate the overall effect of gabapentinoids on hip fractures. We could not conduct subgroup analyses for individual gabapentinoids due to the small number of patients dispensed gabapentin. However, a previous head-to-head study suggested gabapentin and pregabalin were associated with a similar risk of fragility fractures.^52^ While self-controlled design accounts for time-invariant confounders across individuals, the design cannot account for time-varying confounders within individuals (eg, acute pain episodes), so residual confounding is still possible. However, the only reimbursed indication for pregabalin (used by 95% of patients in this study) in Australia is neuropathic pain. It is unlikely that patients experienced substantial progression in overall severity of neuropathic pain across our prespecified assessment periods.^4,64^ While HFRS has been validated for use with administrative data, it is based on *ICD-10-AM* diagnostic codes assigned during hospital episodes; therefore, it may not capture all dimensions of frailty. We also did not have laboratory results, thus we relied on diagnoses recorded in hospitalizations to define CKD. Finally, our study population included residents of 1 state in Australia. While we do not expect that gabapentinoid effects will differ across different countries and populations, caution is needed before transporting our results to other settings, especially those with other indications for gabapentinoids. Future studies in other populations are needed to confirm our findings.

## Conclusions

In this population-based case-case-time-control study of Australian adults hospitalized for first hip fracture, the use of gabapentinoids was associated with a higher risk of hip fractures, especially in patients who were frailer or with chronic kidney disease. In addition to the known risk associated with kidney impairment, frailty status may be an important risk factor when considering use of gabapentinoids.

## Notes

Supplement 1. **eTable 1. **Diagnostic codes for comorbidities **eTable 2. **Characteristics of patients included and matched future-case controls for subgroup analyses for patients with low frailty risk (Hospital Frailty Risk Score [HFRS]<5), high frailty risk (HFRS≥5), without chronic kidney disease (CKD) and with CKD **eTable 3. **Characteristics of patients included and matched future-case controls for sensitivity analyses with exposure assessment period (index and reference periods) of 60 days and washout periods of 90, 120, 150, and 180 days **eTable 4.** Characteristics of patients included and matched future-case controls for sensitivity analyses with exposure assessment period (index and reference periods) of 30 days and washout periods of 30, 60, and 90 days **eTable 5. **Characteristics of patients included and matched future-case controls for sensitivity analyses with exposure assessment period (index and reference periods) of 30 days and washout periods of 120, 150, and 180 days **eTable 6. **Characteristics of patients included and matched future-case controls for sensitivity analyses with exposure assessment period (index and reference periods) of 90 days and washout periods of 90, 120, 150, and 180 days **eTable 7. **Results of case-case-time-control analyses for sensitivity analyses

Supplement 2.
Data Sharing Statement
