# Supplementary material for: Gabapentinoids and Risk of Hip Fracture
Source: JAMA Netw Open. 2024 Nov 13;7(11):e2444488. doi: 10.1001/jamanetworkopen.2024.44488 (PMC11561685; doi:10.1001/jamanetworkopen.2024.44488)
Supplement: Supplement 1. — eTable 1. Diagnostic codes for comorbidities eTable 2. Characteristics of patients included and matched future-case controls for subgroup analyses for patients with low frailty risk (Hospital Frailty Risk Score [HFRS]<5), high frailty risk (HFRS≥5), without chronic kidney disease (CKD) and with CKD eTable 3. Characteristics of patients included and matched future-case controls for sensitivity analyses with exposure assessment period (index and reference periods) of 60 days and washout periods of 90, 120, 150, and 180 days eTable 4. Characteristics of patients included and matched future-case controls for sensitivity analyses with exposure assessment period (index and reference periods) of 30 days and washout periods of 30, 60, and 90 days eTable 5. Characteristics of patients included and matched future-case controls for sensitivity analyses with exposure assessment period (index and reference periods) of 30 days and washout periods of 120, 150, and 180 days eTable 6. Characteristics of patients included and matched future-case controls for sensitivity analyses with exposure assessment period (index and reference periods) of 90 days and washout periods of 90, 120, 150, and 180 days eTable 7. Results of case-case-time-control analyses for sensitivity analyses [file jamanetwopen-e2444488-s001.pdf]

# Supplemental Online Content

Leung MTY, Turner JP, Marquina C, et al. Gabapentinoids and risk of hip fracture. *JAMA Netw Open*. 2024;7(11):e2444488. doi:10.1001/jamanetworkopen.2024.44488

**eTable 1.** Diagnostic codes for comorbidities

**eTable 2.** Characteristics of patients included and matched future-case controls for subgroup analyses for patients with low frailty risk (Hospital Frailty Risk Score [HFRS]<5), high frailty risk (HFRS⩾5), without chronic kidney disease (CKD) and with CKD

**eTable 3.** Characteristics of patients included and matched future-case controls for sensitivity analyses with exposure assessment period (index and reference periods) of 60 days and washout periods of 90, 120, 150, and 180 days

**eTable 4.** Characteristics of patients included and matched future-case controls for sensitivity analyses with exposure assessment period (index and reference periods) of 30 days and washout periods of 30, 60, and 90 days

**eTable 5.** Characteristics of patients included and matched future-case controls for sensitivity analyses with exposure assessment period (index and reference periods) of 30 days and washout periods of 120, 150, and 180 days

**eTable 6.** Characteristics of patients included and matched future-case controls for sensitivity analyses with exposure assessment period (index and reference periods) of 90 days and washout periods of 90, 120, 150, and 180 days

**eTable 7.** Results of case-case-time-control analyses for sensitivity analyses

This supplemental material has been provided by the authors to give readers additional information about their work.

© 2024 Leung MTY et al. *JAMA Network Open*.

**eTable 1.** ICD-10-AM codes for identification of comorbidities.

| Comorbidity | ICD-10-AM |
|---|---|
| Anxiety | F40, F41 |
| Arthritis | M00, M05, M06, M076, M13, M15-M19, M77.2 |
| Alcohol use disorder | F10 |
| Cancer | C00-C99 |
| Cardiovascular disease | I00-I99 |
| Cerebrovascular disease | I60-I69 |
| Chronic kidney disease | N00-19, N25-N28, N39.1, N39.2, D59.3, B52.0, E85.3, Q60-Q63, T82.4, T86.1, Z49.0, Z94.0, Z99.2, U87.1 |
| COPD/ asthma | J40-J46 |
| Delirium | F05 |
| Dementia | F00-F03, F10.7 ,F13.7, F18.7, F05.1, G30 |
| Depression or other affective disorders | F32-F39 |
| Diabetes mellitus | E10, E11, E13, E14, O24 |
| Epilepsy/seizures | G40, R56 |
| Fall | W00-W19 |
| Gastroesophageal reflux disease | K21 |
| Osteoporosis | M80-M82 |
| Peptic ulcer disease | K25-K27 |
| Previous vertebral fracture | M484, S12, S220, S320, M8008 |
| Visual disturbances and blindness | H53, H54 |

**Abbreviation: ICD-10-AM,** International Statistical Classification of Diseases and Related Health Problems, Tenth Revision, Australian Modification

© 2024 Leung MTY et al. *JAMA Network Open*.

**eTable 2.** Characteristics of patients included and matched future-case controls for subgroup analyses for patients with low frailty risk (Hospital Frailty Risk Score [HFRS]<5), high frailty risk (HFRS≥5), without chronic kidney disease (CKD) and with CKD.

| Subgroup | Low frailty risk | | | High frailty risk | | | Without CKD | | | With CKD | | |
|---|---|---|---|---|---|---|---|---|---|---|---|---|
| | Cases | Controls | SMD | Cases | Controls | SMD | Cases | Controls | SMD | Cases | Controls | SMD |
| **Total, n** | 1451 | 6953 | | 1168 | 5502 | | 1822 | 8814 | | 793 | 3688 | |
| **Age, n (%)** | | | 0.098 | | | 0.114 | | | 0.063 | | | 0.146 |
| 50-59 | 62 (4.3) | 178 (2.6) | | 38 (3.3) | 92 (1.7) | | 85 (4.7) | 305 (3.5) | | 13 (1.6) | 23 (0.6) | |
| 60-69 | 170 (11.7) | 776 (11.2) | | 106 (9.1) | 435 (7.9) | | 205 (11.3) | 968 (11.0) | | 70 (8.8) | 226 (6.1) | |
| 70-79 | 369 (25.4) | 1777 (25.6) | | 298 (25.5) | 1401 (25.5) | | 483 (26.5) | 2343 (26.6) | | 186 (23.5) | 857 (23.2) | |
| 80-84 | 281 (19.4) | 1391 (20.0) | | 254 (21.7) | 1262 (22.9) | | 348 (19.1) | 1724 (19.6) | | 187 (23.6) | 927 (25.1) | |
| 85+ | 569 (39.2) | 2831 (40.7) | | 472 (40.4) | 2312 (42.0) | | 701 (38.5) | 3474 (39.4) | | 337 (42.5) | 1655 (44.9) | |
| **Sex, n (%)** | | | 0.035 | | | 0.031 | | | 0.025 | | | 0.022 |
| Males | 369 (25.4) | 1662 (23.9) | | 376 (32.2) | 1692 (30.8) | | 450 (24.7) | 2082 (23.6) | | 289 (36.4) | 1305 (35.4) | |
| Females | 1082 (74.6) | 5291 (76.1) | | 792 (67.8) | 3810 (69.2) | | 1372 (75.3) | 6732 (76.4) | | 504 (63.6) | 2383 (64.6) | |
| **HFRS, n (%)** | | | 0.018 | | | 0.010 | | | 0.030 | | | 0.040 |
| <5 | 518 (35.7( | 2542 (36.6) | | 943 (80.7) | 4420 (80.3) | | 1235 (67.8) | 6097 (69.2) | | 211 (26.6) | 1047 (28.4) | |
| ≥5 | 933 (64.3) | 4411 (63.4) | | 225 (19.3) | 1082 (19.7) | | 587 (32.2) | 2717 (30.8) | | 582 (73.4) | 2641 (71.6) | |
| **Comorbidities, n (%)** | | | | | | | | | | | | |
| Anxiety | 55 (3.8) | 248 (3.6) | 0.012 | 149 (12.8) | 657 (11.9) | 0.025 | 112 (6.1) | 462 (5.2) | 0.039 | 91 (11.5) | 432 (11.7) | 0.007 |
| Arthritis | 249 (17.2) | 1215 (17.5) | 0.008 | 288 (24.7) | 1391 (25.3) | 0.014 | 350 (19.2) | 1725 (19.6) | 0.009 | 187 (23.6) | 861 (23.3) | 0.006 |
| Alcohol use disorder | 27 (1.9) | 79 (1.1) | 0.060 | 69 (5.9) | 281 (5.1) | 0.035 | 58 (3.2) | 245 (2.8) | 0.024 | 38 (4.8) | 163 (4.4) | 0.018 |
| Cancer | 223 (15.4) | 1087 (15.6) | 0.007 | 286 (24.5) | 1468 (26.7) | 0.050 | 326 (17.9) | 1566 (17.8) | 0.003 | 178 (22.4) | 859 (23.3) | 0.020 |
| Cardiovascular disease | 679 (46.8) | 3456 (49.7) | 0.058 | 987 (84.5) | 4642 (84.4) | 0.004 | 977 (53.6) | 4719 (53.5) | 0.002 | 684 (86.3) | 3215 (87.2) | 0.027 |
| Cerebrovascular disease | 87 (6.0) | 445 (6.4) | 0.017 | 182 (15.6) | 842 (15.3) | 0.008 | 145 (8.0) | 725 (8.2) | 0.010 | 123 (15.5) | 518 (14.0) | 0.041 |
| Chronic kidney disease | 214 (14.7) | 1188 (17.1) | 0.064 | 588 (50.3) | 2908 (52.9) | 0.050 | 1822 (100.0) | 8814 (100.0) | <0.001 | 793 (100.0) | 3688 (100.0) | <0.001 |
| COPD/ asthma | 122 (8.4) | 595 (8.6) | 0.005 | 250 (21.4) | 1098 (20.0) | 0.036 | 185 (10.2) | 783 (8.9) | 0.043 | 183 (23.1) | 881 (23.9) | 0.019 |
| Delirium | 78 (5.4) | 407 (5.9) | 0.021 | 343 (29.4) | 1604 (29.2) | 0.005 | 185 (10.2) | 920 (10.4) | 0.009 | 234 (29.5) | 1021 (27.7) | 0.040 |
| Dementia | 25 (1.7) | 109 (1.6) | 0.012 | 91 (7.8) | 468 (8.5) | 0.026 | 57 (3.1) | 293 (3.3) | 0.011 | 58 (7.3) | 287 (7.8) | 0.018 |

© 2024 Leung MTY et al. *JAMA Network Open*.

| | | | | | | | | | | | | |
|---|---|---|---|---|---|---|---|---|---|---|---|---|
| Depression or other affective disorders | 38 (2.6) | 159 (2.3) | 0.021 | 133 (11.4) | 633 (11.5) | 0.004 | 89 (4.9) | 418 (4.7) | 0.007 | 83 (10.5) | 381 (10.3) | 0.004 |
| Diabetes mellitus | 284 (19.6) | 1355 (19.5) | 0.002 | 412 (35.3) | 1952 (35.5) | 0.004 | 318 (17.5) | 1605 (18.2) | 0.020 | 374 (47.2) | 1680 (45.6) | 0.032 |
| Epilepsy/seizures | 23 (1.6) | 82 (1.2) | 0.035 | 50 (4.3) | 225 (4.1) | 0.010 | 40 (2.2) | 179 (2.0) | 0.011 | 33 (4.2) | 143 (3.9) | 0.014 |
| Fall | 250 (17.2) | 1152 (16.6) | 0.018 | 621 (53.2) | 3056 (55.5) | 0.048 | 514 (28.2) | 2435 (27.6) | 0.013 | 357 (45.0) | 1751 (47.5) | 0.049 |
| Gastroesophageal reflux disease | 106 (7.3) | 536 (7.7) | 0.015 | 152 (13.0) | 618 (11.2) | 0.055 | 162 (8.9) | 790 (9.0) | 0.003 | 94 (11.9) | 413 (11.2) | 0.021 |
| Osteoporosis | 312 (21.5) | 1536 (22.1) | 0.014 | 455 (39.0) | 2184 (39.7) | 0.015 | 481 (26.4) | 2426 (27.5) | 0.025 | 283 (35.7) | 1235 (33.5) | 0.046 |
| Peptic ulcer disease | 34 (2.3) | 158 (2.3) | 0.005 | 64 (5.5) | 279 (5.1) | 0.018 | 44 (2.4) | 199 (2.3) | 0.010 | 52 (6.6) | 185 (5.0) | 0.066 |
| Previous vertebral fracture | 31 (2.1) | 134 (1.9) | 0.015 | 123 (10.5) | 731 (13.3) | 0.085 | 88 (4.8) | 422 (4.8) | 0.002 | 67 (8.4) | 377 (10.2) | 0.061 |
| Visual disturbances and blindness | 28 (1.9) | 136 (2.0) | 0.002 | 60 (5.1) | 272 (4.9) | 0.009 | 49 (2.7) | 241 (2.7) | 0.003 | 37 (4.7) | 182 (4.9) | 0.013 |

**Abbreviations:** COPD, chronic obstructive pulmonary disease; SMD, standardized mean difference

© 2024 Leung MTY et al. *JAMA Network Open*.

**eTable 3.** Characteristics of patients included and matched future-case controls for sensitivity analyses with exposure assessment period (index and reference periods) of 60 days and washout periods of 90, 120, 150, and 180 days.

| **Washout period** | **90 days** | | | **120 days** | | | **150 days** | | | **180 days** | | |
|---|---|---|---|---|---|---|---|---|---|---|---|---|
| | **Cases** | **Controls** | **SMD** | **Cases** | **Controls** | **SMD** | **Cases** | **Controls** | **SMD** | **Cases** | **Controls** | **SMD** |
| **Total, n** | 2630 | 12846 | | 2632 | 12844 | | 2630 | 12852 | | 2615 | 12786 | |
| **Age, n (%)** | | | 0.04 | | | 0.039 | | | 0.04 | | | 0.041 |
| 50-59 | 110 (4.2) | 442 (3.4) | | 111 (4.2) | 448 (3.5) | | 112 (4.3) | 451 (3.5) | | 113 (4.3) | 453 (3.5) | |
| 60-69 | 278 (10.6) | 1350 (10.5) | | 282 (10.7) | 1359 (10.6) | | 282 (10.7) | 1365 (10.6) | | 280 (10.7) | 1369 (10.7) | |
| 70-79 | 669 (25.4) | 3257 (25.4) | | 663 (25.2) | 3226 (25.1) | | 653 (24.8) | 3190 (24.8) | | 657 (25.1) | 3208 (25.1) | |
| 80-84 | 533 (20.3) | 2646 (20.6) | | 539 (20.5) | 2672 (20.8) | | 535 (20.3) | 2652 (20.6) | | 535 (20.5) | 2652 (20.7) | |
| 85+ | 1040 (39.5) | 5151 (40.1) | | 1037 (39.4) | 5139 (40.0) | | 1048 (39.8) | 5194 (40.4) | | 1030 (39.4) | 5104 (39.9) | |
| **Sex, n (%)** | | | 0.019 | | | 0.021 | | | 0.018 | | | 0.017 |
| Males | 754 (28.7) | 3571 (27.8) | | 764 (29.0) | 3609 (28.1) | | 764 (29.0) | 3626 (28.2) | | 760 (29.1) | 3615 (28.3) | |
| Females | 1876 (71.3) | 9275 (72.2) | | 1868 (71.0) | 9235 (71.9) | | 1866 (71.0) | 9226 (71.8) | | 1855 (70.9) | 9171 (71.7) | |
| **HFRS, n (%)** | | | 0.001 | | | 0.005 | | | 0.031 | | | 0.020 |
| <5 | 1447 (55.0) | 7062 (55.0) | | 1183 (54.7) | 7062 (55.0) | | 1433 (54.5) | 7198 (56.0) | | 1434 (54.4) | 7085 (55.4) | |
| ≥5 | 1183 (45.0) | 5784 (45.0) | | 1191 (45.3) | 5782 (45.0) | 0.005 | 1197 (45.5) | 5654 (44.0) | 0.031 | 1192 (45.6) | 5701 (44.6) | 0.020 |
| **Comorbidities, n (%)** | | | | | | | | | | | | |
| Anxiety | 210 (8.0) | 977 (7.6) | 0.014 | 209 (7.9) | 1040 (8.1) | 0.006 | 212 (8.1) | 1033 (8.0) | 0.001 | 206 (7.9) | 997 (7.8) | 0.003 |
| Arthritis | 538 (20.5) | 2717 (21.2) | 0.017 | 546 (20.7) | 2686 (20.9) | 0.004 | 544 (20.7) | 2718 (21.1) | 0.011 | 542 (20.7) | 2646 (20.7) | 0.001 |
| Alcohol use disorder | 100 (3.8) | 476 (3.7) | 0.005 | 102 (3.9) | 439 (3.4) | 0.024 | 103 (3.9) | 456 (3.5) | 0.019 | 103 (3.9) | 458 (3.6) | 0.019 |
| Cancer | 506 (19.2) | 2541 (19.8) | 0.014 | 514 (19.5) | 2502 (19.5) | 0.001 | 507 (19.3) | 2633 (20.5) | 0.030 | 505 (19.3) | 2560 (20.0) | 0.018 |
| Cardiovascular disease | 1679 (63.8) | 8365 (65.1) | 0.027 | 1686 (64.1) | 8338 (64.9) | 0.018 | 1688 (64.2) | 8302 (64.6) | 0.009 | 1683 (64.4) | 8297 (64.9) | 0.011 |
| Cerebrovascular disease | 276 (10.5) | 1393 (10.8) | 0.011 | 275 (10.4) | 1376 (10.7) | 0.009 | 276 (10.5) | 1334 (10.4) | 0.004 | 277 (10.6) | 1290 (10.1) | 0.017 |
| Chronic kidney disease | 806 (30.6) | 4170 (32.5) | 0.039 | 816 (31.0) | 4203 (32.7) | 0.037 | 820 (31.2) | 4153 (32.3) | 0.024 | 813 (31.1) | 4199 (32.8) | 0.038 |
| COPD/ asthma | 375 (14.3) | 1795 (14.0) | 0.008 | 378 (14.4) | 1848 (14.4) | 0.001 | 383 (14.6) | 1815 (14.1) | 0.013 | 380 (14.5) | 1872 (14.6) | 0.003 |
| Delirium | 421 (16.0) | 2116 (16.5) | 0.013 | 422 (16.0) | 2176 (16.9) | 0.024 | 426 (16.2) | 2104 (16.4) | 0.005 | 426 (16.3) | 2119 (16.6) | 0.008 |

© 2024 Leung MTY et al. *JAMA Network Open*.

| | | | | | | | | | | | | |
|---|---|---|---|---|---|---|---|---|---|---|---|---|
| Dementia | 109 (4.1) | 599 (4.7) | 0.025 | 111 (4.2) | 675 (5.3) | 0.049 | 114 (4.3) | 595 (4.6) | 0.014 | 112 (4.3) | 611 (4.8) | 0.024 |
| Depression or other affective disorders | 176 (6.7) | 874 (6.8) | 0.004 | 175 (6.6) | 897 (7.0) | 0.013 | 177 (6.7) | 902 (7.0) | 0.011 | 179 (6.8) | 874 (6.8) | <0.001 |
| Diabetes mellitus | 698 (26.5) | 3425 (26.7) | 0.003 | 695 (26.4) | 3495 (27.2) | 0.018 | 700 (26.6) | 3419 (26.6) | <0.001 | 704 (26.9) | 3534 (27.6) | 0.016 |
| Epilepsy/seizures | 78 (3.0) | 312 (2.4) | 0.033 | 77 (2.9) | 356 (2.8) | 0.009 | 76 (2.9) | 357 (2.8) | 0.007 | 74 (2.8) | 329 (2.6) | 0.016 |
| Fall | 877 (33.3) | 4385 (34.1) | 0.017 | 878 (33.4) | 4418 (34.4) | 0.022 | 884 (33.6) | 4392 (34.2) | 0.012 | 887 (33.9) | 4444 (34.8) | 0.018 |
| Gastroesophageal reflux disease | 261 (9.9) | 1231 (9.6) | 0.012 | 261 (9.9) | 1191 (9.3) | 0.022 | 262 (10.0) | 1253 (9.7) | 0.007 | 260 (9.9) | 1213 (9.5) | 0.015 |
| Osteoporosis | 778 (29.6) | 3753 (29.2) | 0.008 | 780 (29.6) | 3747 (29.2) | 0.010 | 776 (29.5) | 3878 (30.2) | 0.015 | 773 (29.6) | 3803 (29.7) | 0.004 |
| Peptic ulcer disease | 99 (3.8) | 451 (3.5) | 0.014 | 99 (3.8) | 452 (3.5) | 0.013 | 98 (3.7) | 419 (3.3) | 0.025 | 98 (3.7) | 444 (3.5) | 0.015 |
| Previous vertebral fracture | 158 (6.0) | 894 (7.0) | 0.039 | 161 (6.1) | 872 (6.8) | 0.027 | 164 (6.2) | 859 (6.7) | 0.018 | 164 (6.3) | 928 (7.3) | 0.039 |
| Visual disturbances and blindness | 89 (3.4) | 464 (3.6) | 0.012 | 90 (3.4) | 411 (3.2) | 0.012 | 90 (3.4) | 451 (3.5) | 0.005 | 90 (3.4) | 418 (3.3) | 0.010 |

**Abbreviations:** COPD, chronic obstructive pulmonary disease; SMD, standardized mean difference

© 2024 Leung MTY et al. *JAMA Network Open*.

**eTable 4.** Characteristics of patients included and matched future-case controls for sensitivity analyses with exposure assessment period (index and reference periods) of 30 days and washout periods of 30, 60, and 90 days.

| Washout period | 30 days | | | 60 days | | | 90 days | | |
|---|---|---|---|---|---|---|---|---|---|
| | Cases | Controls | SMD | Cases | Controls | SMD | Cases | Controls | SMD |
| **Total, n** | 2660 | 12980 | | 2643 | 12909 | | 2649 | 12930 | |
| **Age, n (%)** | | | 0.043 | | | 0.04 | | | 0.039 |
| 50-59 | 113 (4.2) | 446 (3.4) | | 114 (4.3) | 457 (3.5) | | 108 (4.1) | 434 (3.4) | |
| 60-69 | 291 (10.9) | 1413 (10.9) | | 285 (10.8) | 1379 (10.7) | | 289 (10.9) | 1399 (10.8) | |
| 70-79 | 674 (25.3) | 3283 (25.3) | | 666 (25.2) | 3264 (25.3) | | 671 (25.3) | 3262 (25.2) | |
| 80-84 | 535 (20.1) | 2652 (20.4) | | 534 (20.2) | 2647 (20.5) | | 530 (20.0) | 2629 (20.3) | |
| 85+ | 1047 (39.4) | 5186 (40.0) | | 1044 (39.5) | 5162 (40.0) | | 1051 (39.7) | 5206 (40.3) | |
| **Sex, n (%)** | | | 0.02 | | | 0.018 | | | 0.019 |
| Males | 769 (28.9) | 3636 (28.0) | | 763 (28.9) | 3623 (28.1) | | 764 (28.8) | 3621 (28.0) | |
| Females | 1891 (71.1) | 9344 (72.0) | | 1880 (71.1) | 9286 (71.9) | | 1885 (71.2) | 9309 (72.0) | |
| **HFRS, n (%)** | | | 0.022 | | | 0.01 | | | 0.021 |
| <5 | 1470 (55.3) | 7031 (54.2) | | 1459 (55.2) | 7190 (55.7) | | 1451 (54.8) | 7216 (55.8) | |
| ≥5 | 1190 (44.7) | 5949 (45.8) | 0.022 | 1184 (44.8) | 5719 (44.3) | 0.01 | 1198 (45.2) | 5714 (44.2) | 0.021 |
| **Comorbidities, n (%)** | | | | | | | | | |
| Anxiety | 210 (7.9) | 1013 (7.8) | 0.003 | 209 (7.9) | 1034 (8.0) | 0.004 | 205 (7.7) | 961 (7.4) | 0.012 |
| Arthritis | 530 (19.9) | 2631 (20.3) | 0.009 | 538 (20.4) | 2685 (20.8) | 0.011 | 542 (20.5) | 2721 (21.0) | 0.014 |
| Alcohol use disorder | 105 (3.9) | 464 (3.6) | 0.020 | 103 (3.9) | 474 (3.7) | 0.012 | 103 (3.9) | 461 (3.6) | 0.017 |
| Cancer | 514 (19.3) | 2577 (19.9) | 0.013 | 506 (19.1) | 2523 (19.5) | 0.010 | 510 (19.3) | 2594 (20.1) | 0.020 |
| Cardiovascular disease | 1700 (63.9) | 8490 (65.4) | 0.031 | 1683 (63.7) | 8388 (65.0) | 0.027 | 1687 (63.7) | 8315 (64.3) | 0.013 |
| Cerebrovascular disease | 276 (10.4) | 1372 (10.6) | 0.006 | 274 (10.4) | 1312 (10.2) | 0.007 | 279 (10.5) | 1284 (9.9) | 0.020 |
| Chronic kidney disease | 815 (30.6) | 4342 (33.5) | 0.060 | 805 (30.5) | 4254 (33.0) | 0.054 | 816 (30.8) | 4177 (32.3) | 0.032 |
| COPD/ asthma | 380 (14.3) | 1931 (14.9) | 0.017 | 372 (14.1) | 1892 (14.7) | 0.017 | 370 (14.0) | 1821 (14.1) | 0.003 |
| Delirium | 424 (15.9) | 2168 (16.7) | 0.021 | 424 (16.0) | 2120 (16.4) | 0.010 | 427 (16.1) | 2142 (16.6) | 0.012 |
| Dementia | 111 (4.2) | 635 (4.9) | 0.035 | 114 (4.3) | 645 (5.0) | 0.032 | 116 (4.4) | 611 (4.7) | 0.017 |
| Depression or other affective disorders | 176 (6.6) | 862 (6.6) | 0.001 | 172 (6.5) | 857 (6.6) | 0.005 | 173 (6.5) | 836 (6.5) | 0.003 |
| Diabetes mellitus | 700 (26.3) | 3483 (26.8) | 0.012 | 700 (26.5) | 3555 (27.5) | 0.024 | 705 (26.6) | 3501 (27.1) | 0.010 |
| Epilepsy/seizures | 80 (3.0) | 356 (2.7) | 0.016 | 76 (2.9) | 326 (2.5) | 0.022 | 79 (3.0) | 327 (2.5) | 0.028 |
| Fall | 882 (33.2) | 4472 (34.5) | 0.027 | 880 (33.3) | 4358 (33.8) | 0.010 | 884 (33.4) | 4344 (33.6) | 0.005 |
| Gastroesophageal reflux disease | 265 (10.0) | 1255 (9.7) | 0.010 | 260 (9.8) | 1250 (9.7) | 0.005 | 262 (9.9) | 1245 (9.6) | 0.009 |
| Osteoporosis | 771 (29.0) | 3821 (29.4) | 0.010 | 769 (29.1) | 3738 (29.0) | 0.003 | 777 (29.3) | 3779 (29.2) | 0.002 |
| Peptic ulcer disease | 103 (3.9) | 457 (3.5) | 0.019 | 100 (3.8) | 416 (3.2) | 0.031 | 100 (3.8) | 415 (3.2) | 0.031 |
| Previous vertebral fracture | 166 (6.2) | 918 (7.1) | 0.033 | 164 (6.2) | 863 (6.7) | 0.020 | 156 (5.9) | 856 (6.6) | 0.030 |
| Visual disturbances and blindness | 97 (3.6) | 448 (3.5) | 0.011 | 95 (3.6) | 417 (3.2) | 0.020 | 96 (3.6) | 464 (3.6) | 0.002 |

**Abbreviations:** COPD, chronic obstructive pulmonary disease; SMD, standardized mean difference

© 2024 Leung MTY et al. *JAMA Network Open*.

**eTable 5.** Characteristics of patients included and matched future-case controls for sensitivity analyses with exposure assessment period (index and reference periods) of 30 days and washout periods of 120, 150, and 180 days.

| Washout period | 120 days | | | 150 days | | | 180 days | | |
|---|---|---|---|---|---|---|---|---|---|
| | Cases | Controls | SMD | Cases | Controls | SMD | Cases | Controls | SMD |
| **Total, n** | 2652 | 12959 | | 2639 | 12901 | | 2613 | 12774 | |
| **Age, n (%)** | | | 0.04 | | | 0.041 | | | 0.038 |
| 50-59 | 107 (4.1) | 429 (3.4) | | 108 (4.1) | 431 (3.4) | | 109 (4.2) | 441 (3.4) | |
| 60-69 | 281 (10.8) | 1375 (10.8) | | 285 (10.9) | 1380 (10.8) | | 286 (10.9) | 1384 (10.8) | |
| 70-79 | 662 (25.4) | 3226 (25.3) | | 668 (25.5) | 3251 (25.4) | | 657 (25.1) | 3205 (25.0) | |
| 80-84 | 524 (20.1) | 2607 (20.4) | | 530 (20.2) | 2627 (20.5) | | 532 (20.3) | 2637 (20.6) | |
| 85+ | 1037 (39.7) | 5136 (40.2) | | 1030 (39.3) | 5106 (39.9) | | 1037 (39.6) | 5139 (40.1) | |
| **Sex, n (%)** | | | 0.017 | | | 0.020 | | | 0.018 |
| Males | 746 (28.6) | 3550 (27.8) | | 755 (28.8) | 3573 (27.9) | | 757 (28.9) | 3593 (28.1) | |
| Females | 1865 (71.4) | 9223 (72.2) | | 1866 (71.2) | 9222 (72.1) | | 1864 (71.1) | 9213 (71.9) | |
| **HFRS, n (%)** | | | 0.016 | | | 0.008 | | | 0.019 |
| <5 | 1448 (55.5) | 7186 (56.3) | | 1141 (55.0) | 7085 (55.4) | | 1437 (54.8) | 7139 (55.7) | |
| ≥5 | 1163 (44.5) | 5587 (43.7) | | 1180 (45.0) | 5710 (44.6) | | 1184 (45.2) | 5667 (44.3) | |
| **Comorbidities, n (%)** | | | | | | | | | |
| Anxiety | 205 (7.9) | 934 (7.3) | 0.020 | 203 (7.7) | 970 (7.6) | 0.006 | 206 (7.9) | 1008 (7.9) | <0.001 |
| Arthritis | 536 (20.5) | 2696 (21.1) | 0.014 | 534 (20.4) | 2687 (21.0) | 0.015 | 536 (20.5) | 2664 (20.8) | 0.009 |
| Alcohol use disorder | 99 (3.8) | 420 (3.3) | 0.027 | 101 (3.9) | 442 (3.5) | 0.021 | 101 (3.9) | 425 (3.3) | 0.029 |
| Cancer | 499 (19.1) | 2539 (19.9) | 0.019 | 508 (19.4) | 2591 (20.3) | 0.022 | 506 (19.3) | 2543 (19.9) | 0.014 |
| Cardiovascular disease | 1659 (63.5) | 8178 (64.0) | 0.010 | 1675 (63.9) | 8274 (64.7) | 0.016 | 1674 (63.9) | 8250 (64.4) | 0.012 |
| Cerebrovascular disease | 273 (10.5) | 1262 (9.9) | 0.019 | 276 (10.5) | 1363 (10.7) | 0.004 | 278 (10.6) | 1296 (10.1) | 0.016 |
| Chronic kidney disease | 798 (30.6) | 4093 (32.0) | 0.032 | 805 (30.7) | 4164 (32.5) | 0.039 | 817 (31.2) | 4028 (31.5) | 0.006 |
| COPD/ asthma | 367 (14.1) | 1842 (14.4) | 0.010 | 368 (14.0) | 1789 (14.0) | 0.002 | 370 (14.1) | 1828 (14.3) | 0.005 |
| Delirium | 416 (15.9) | 2050 (16.0) | 0.003 | 413 (15.8) | 2066 (16.1) | 0.011 | 423 (16.1) | 2085 (16.3) | 0.004 |
| Dementia | 110 (4.2) | 599 (4.7) | 0.023 | 108 (4.1) | 580 (4.5) | 0.020 | 114 (4.3) | 603 (4.7) | 0.017 |
| Depression or other affective disorders | 171 (6.5) | 839 (6.6) | 0.001 | 171 (6.5) | 847 (6.6) | 0.004 | 177 (6.8) | 871 (6.8) | 0.002 |
| Diabetes mellitus | 690 (26.4) | 3388 (26.5) | 0.002 | 689 (26.3) | 3479 (27.2) | 0.020 | 695 (26.5) | 3397 (26.5) | <0.001 |
| Epilepsy/seizures | 74 (2.8) | 315 (2.5) | 0.023 | 77 (2.9) | 327 (2.6) | 0.023 | 77 (2.9) | 332 (2.6) | 0.021 |
| Fall | 865 (33.1) | 4283 (33.5) | 0.009 | 868 (33.1) | 4368 (34.1) | 0.022 | 873 (33.3) | 4320 (33.7) | 0.009 |
| Gastroesophageal reflux disease | 258 (9.9) | 1233 (9.7) | 0.008 | 262 (10.0) | 1202 (9.4) | 0.020 | 262 (10.0) | 1224 (9.6) | 0.015 |
| Osteoporosis | 763 (29.2) | 3772 (29.5) | 0.007 | 776 (29.6) | 3831 (29.9) | 0.007 | 775 (29.6) | 3779 (29.5) | 0.001 |
| Peptic ulcer disease | 101 (3.9) | 442 (3.5) | 0.022 | 99 (3.8) | 438 (3.4) | 0.019 | 99 (3.8) | 423 (3.3) | 0.026 |
| Previous vertebral fracture | 151 (5.8) | 903 (7.1) | 0.052 | 161 (6.1) | 887 (6.9) | 0.032 | 161 (6.1) | 871 (6.8) | 0.027 |
| Visual disturbances and blindness | 91 (3.5) | 426 (3.3) | 0.008 | 94 (3.6) | 452 (3.5) | 0.003 | 92 (3.5) | 465 (3.6) | 0.007 |

**Abbreviations:** COPD, chronic obstructive pulmonary disease; SMD, standardized mean difference

© 2024 Leung MTY et al. *JAMA Network Open*.

**eTable 6.** Characteristics of patients included and matched future-case controls for sensitivity analyses with exposure assessment period (index and reference periods) of 90 days and washout periods of 90, 120, 150, and 180 days.

| **Washout period** | **90 days** | | | **120 days** | | | **150 days** | | | **180 days** | | |
|---|---|---|---|---|---|---|---|---|---|---|---|---|
| | **Cases** | **Controls** | **SMD** | **Cases** | **Controls** | **SMD** | **Cases** | **Controls** | **SMD** | **Cases** | **Controls** | **SMD** |
| **Total, n** | 2652 | 12959 | | 2639 | 12901 | | 2613 | 12774 | | 2578 | 12609 | |
| **Age, n (%)** | | | 0.039 | | | 0.038 | | | 0.038 | | | 0.034 |
| 50-59 | 113 (4.3) | 457 (3.5) | | 112 (4.2) | 454 (3.5) | | 111 (4.2) | 451 (3.5) | | 109 (4.2) | 452 (3.6) | |
| 60-69 | 288 (10.9) | 1394 (10.8) | | 285 (10.8) | 1382 (10.7) | | 286 (10.9) | 1387 (10.9) | | 282 (10.9) | 1367 (10.8) | |
| 70-79 | 656 (24.7) | 3202 (24.7) | | 655 (24.8) | 3199 (24.8) | | 646 (24.7) | 3155 (24.7) | | 639 (24.8) | 3119 (24.7) | |
| 80-84 | 540 (20.4) | 2677 (20.7) | | 541 (20.5) | 2682 (20.8) | | 540 (20.7) | 2677 (21.0) | | 533 (20.7) | 2642 (21.0) | |
| 85+ | 1055 (39.8) | 5229 (40.4) | | 1046 (39.6) | 5184 (40.2) | | 1030 (39.4) | 5104 (40.0) | | 1015 (39.4) | 5029 (39.9) | |
| **Sex, n (%)** | | | 0.018 | | | 0.018 | | | 0.017 | | | 0.017 |
| Males | 765 (28.8) | 3631 (28.0) | | 761 (28.8) | 3615 (28.0) | | 759 (29.0) | 3610 (28.3) | | 749 (29.1) | 3564 (28.3) | |
| Females | 1887 (71.2) | 9328 (72.0) | | 1878 (71.2) | 9286 (72.0) | | 1854 (71.0) | 9164 (71.7) | | 1829 (70.9) | 9045 (71.7) | |
| **HFRS, n (%)** | | | 0.009 | | | 0.001 | | | 0.019 | | | 0.026 |
| <5 | 1436 (54.1) | 7067 (54.6) | | 1426 (54.0) | 6964 (54.0) | | 1410 (54.0) | 7017 (54.9) | | 1392 (54.0) | 6970 (55.3) | |
| ≥5 | 1216 (45.9) | 5883 (45.4) | | 1213 (46.0) | 5937 (46.0) | | 1203 (46.0) | 5757 (45.1) | | 1186 (46.0) | 5639 (44.7) | |
| **Comorbidities, n (%)** | | | | | | | | | | | | |
| Anxiety | 212 (8.0) | 1004 (7.7) | 0.009 | 211 (8.0) | 990 (7.7) | 0.012 | 203 (7.8) | 995 (7.8) | 0.001 | 199 (7.7) | 975 (7.7) | 0.001 |
| Arthritis | 553 (20.9) | 2747 (21.2) | 0.008 | 554 (21.0) | 2664 (20.6) | 0.008 | 544 (20.8) | 2702 (21.2) | 0.008 | 538 (20.9) | 2709 (21.5) | 0.015 |
| Alcohol use disorder | 102 (3.8) | 465 (3.6) | 0.014 | 103 (3.9) | 452 (3.5) | 0.021 | 102 (3.9) | 463 (3.6) | 0.015 | 98 (3.8) | 433 (3.4) | 0.020 |
| Cancer | 519 (19.6) | 2627 (20.3) | 0.018 | 517 (19.6) | 2581 (20.0) | 0.010 | 513 (19.6) | 2533 (19.8) | 0.005 | 504 (19.6) | 2492 (19.8) | 0.005 |
| Cardiovascular disease | 1705 (64.3) | 8459 (65.3) | 0.021 | 1696 (64.3) | 8400 (65.1) | 0.018 | 1681 (64.3) | 8256 (64.6) | 0.006 | 1655 (64.2) | 8123 (64.4) | 0.005 |
| Cerebrovascular disease | 273 (10.3) | 1332 (10.3) | 0.001 | 273 (10.3) | 1343 (10.4) | 0.002 | 273 (10.4) | 1324 (10.4) | 0.003 | 270 (10.5) | 1251 (9.9) | 0.018 |
| Chronic kidney disease | 827 (31.2) | 4228 (32.6) | 0.031 | 824 (31.2) | 4317 (33.5) | 0.048 | 822 (31.5) | 4152 (32.5) | 0.022 | 812 (31.5) | 4191 (33.2) | 0.037 |
| COPD/ asthma | 382 (14.4) | 1862 (14.4) | 0.001 | 385 (14.6) | 1874 (14.5) | 0.002 | 382 (14.6) | 1840 (14.4) | 0.006 | 375 (14.5) | 1789 (14.2) | 0.010 |
| Delirium | 424 (16.0) | 2166 (16.7) | 0.020 | 421 (16.0) | 2209 (17.1) | 0.031 | 418 (16.0) | 2142 (16.8) | 0.021 | 414 (16.1) | 2090 (16.6) | 0.014 |
| Dementia | 113 (4.3) | 614 (4.7) | 0.023 | 113 (4.3) | 635 (4.9) | 0.031 | 113 (4.3) | 597 (4.7) | 0.017 | 115 (4.5) | 575 (4.6) | 0.005 |
| Depression or other affective disorders | 178 (6.7) | 930 (7.2) | 0.018 | 182 (6.9) | 916 (7.1) | 0.008 | 176 (6.7) | 949 (7.4) | 0.027 | 178 (6.9) | 906 (7.2) | 0.011 |
| Diabetes mellitus | 707 (26.7) | 3513 (27.1) | 0.010 | 711 (26.9) | 3591 (27.8) | 0.020 | 710 (27.2) | 3421 (26.8) | 0.009 | 705 (27.3) | 3465 (27.5) | 0.003 |
| Epilepsy/seizures | 77 (2.9) | 357 (2.8) | 0.009 | 76 (2.9) | 375 (2.9) | 0.002 | 74 (2.8) | 340 (2.7) | 0.010 | 76 (2.9) | 329 (2.6) | 0.021 |
| Fall | 895 (33.7) | 4431 (34.2) | 0.009 | 898 (34.0) | 4538 (35.2) | 0.024 | 887 (33.9) | 4411 (34.5) | 0.012 | 866 (33.6) | 4250 (33.7) | 0.002 |
| Gastroesophageal reflux disease | 264 (10.0) | 1226 (9.5) | 0.017 | 263 (10.0) | 1198 (9.3) | 0.023 | 259 (9.9) | 1235 (9.7) | 0.008 | 256 (9.9) | 1261 (10.0) | 0.002 |
| Osteoporosis | 788 (29.7) | 3853 (29.7) | <0.001 | 785 (29.7) | 3882 (30.1) | 0.008 | 779 (29.8) | 3790 (29.7) | 0.003 | 762 (29.6) | 3747 (29.7) | 0.003 |
| Peptic ulcer disease | 101 (3.8) | 431 (3.3) | 0.026 | 99 (3.8) | 417 (3.2) | 0.028 | 97 (3.7) | 425 (3.3) | 0.021 | 95 (3.7) | 430 (3.4) | 0.015 |

© 2024 Leung MTY et al. *JAMA Network Open*.

| | | | | | | | | | | | | |
|---|---|---|---|---|---|---|---|---|---|---|---|---|
| Previous vertebral fracture | 169 (6.4) | 954 (7.4) | 0.039 | 169 (6.4) | 922 (7.1) | 0.030 | 168 (6.4) | 888 (7.0) | 0.021 | 163 (6.3) | 881 (7.0) | 0.027 |
| Visual disturbances and blindness | 91 (3.4) | 466 (3.6) | 0.009 | 93 (3.5) | 473 (3.7) | 0.008 | 92 (3.5) | 446 (3.5) | 0.002 | 93 (3.6) | 436 (3.5) | 0.008 |

**Abbreviations:** COPD, chronic obstructive pulmonary disease; SMD, standardized mean difference

© 2024 Leung MTY et al. *JAMA Network Open*.

**eTable 7.** Results of case-case-time-control analyses for sensitivity analyses.

| Exposure assessment period | 60 days | | | 30 days | | | 90 days | | |
|---|---|---|---|---|---|---|---|---|---|
| | Index period only | Reference period only | Odds ratio (95% CI) | Index period only | Reference period only | Odds ratio (95% CI) | Index period only | Reference period only | Odds ratio (95% CI) |
| **Washout period: 30 days** | | | | | | | | | |
| Case crossover | | | | 362 | 197 | 1.88 [1.58-2.24] | | | |
| Control crossover | | | | 880 | 686 | 1.32 [1.20-1.45] | | | |
| Case-case-time-control | | | | N.A. | N.A. | 1.42 [1.17-1.74] | | | |
| Adjusted case-case-time-control[a] | | | | N.A. | N.A. | 1.40 [1.15-1.71] | | | |
| **Washout period: 60 days** | | | | | | | | | |
| Case crossover | | | | 416 | 232 | 1.79 [1.53-2.11] | 467 | 235 | 1.99 [1.70-2.32] |
| Control crossover | | | | 1010 | 860 | 1.17 [1.07-1.29] | 1472 | 1089 | 1.35 [1.25-1.46] |
| Case-case-time-control | | | | N.A. | N.A. | 1.53 [1.27-1.84] | N.A. | N.A. | 1.47 [1.23-1.75] |
| Adjusted case-case-time-control[a] | | | | N.A. | N.A. | 1.50 [1.24-1.80] | N.A. | N.A. | 1.43 [1.20-1.70] |
| **Washout period: 90 days** | | | | | | | | | |
| Case crossover | 459 | 224 | 2.05 [1.75-2.40] | 426 | 227 | 1.88 [1.60-2.20] | 508 | 261 | 1.95 [1.68-2.26] |
| Control crossover | 1496 | 1076 | 1.39 [1.29-1.50] | 1255 | 1005 | 1.25 [1.15-1.36] | 1805 | 1386 | 1.30 [1.21-1.40] |
| Case-case-time-control | N.A. | N.A. | 1.47 [1.23-1.76] | N.A. | N.A. | 1.50 [1.25-1.80] | N.A. | N.A. | 1.49 [1.27-1.76] |
| Adjusted case-case-time-control[a] | N.A. | N.A. | 1.42 [1.19-1.70] | N.A. | N.A. | 1.46 [1.22-1.75] | N.A. | N.A. | 1.45 [1.23-1.72] |
| **Washout period: 120 days** | | | | | | | | | |
| Case crossover | 485 | 238 | 2.04 [1.75-2.38] | 484 | 228 | 2.12 [1.81-2.48] | 540 | 263 | 2.05 [1.77-2.38] |
| Control crossover | 1697 | 1305 | 1.30 [1.21-1.40] | 1402 | 976 | 1.44 [1.32-1.56] | 2109 | 1326 | 1.59 [1.48-1.70] |
| Case-case-time-control | N.A. | N.A. | 1.57 [1.32-1.86] | N.A. | N.A. | 1.48 [1.24-1.76] | N.A. | N.A. | 1.29 [1.10-1.52] |
| Adjusted case-case-time-control[a] | N.A. | N.A. | 1.53 [1.28-1.81] | N.A. | N.A. | 1.44 [1.21-1.72] | N.A. | N.A. | 1.26 [1.06-1.48] |
| **Washout period: 150 days** | | | | | | | | | |
| Case crossover | 522 | 258 | 2.02 [1.74-2.35] | 494 | 239 | 2.07 [1.77-2.41] | 556 | 268 | 2.07 [1.79-2.4] |
| Control crossover | 1895 | 1413 | 1.34 [1.25-1.44] | 1679 | 1213 | 1.38 [1.29-1.49] | 2341 | 1626 | 1.44 [1.35-1.53] |
| Case-case-time-control | N.A. | N.A. | 1.51 [1.28-1.78] | N.A. | N.A. | 1.49 [1.26-1.77] | N.A. | N.A. | 1.44 [1.23-1.69] |
| Adjusted case-case-time-control[a] | N.A. | N.A. | 1.46 [1.24-1.73] | N.A. | N.A. | 1.43 [1.21-1.70] | N.A. | N.A. | 1.40 [1.19-1.64] |
| **Washout period: 180 days** | | | | | | | | | |
| Case crossover | 549 | 258 | 2.13 [1.84-2.47] | 514 | 243 | 2.12 [1.82-2.46] | 580 | 267 | 2.17 [1.88-2.51] |
| Control crossover | 2160 | 1431 | 1.51 [1.41-1.61] | 1738 | 1320 | 1.32 [1.23-1.41] | 2296 | 1565 | 1.47 [1.38-1.56] |
| Case-case-time-control | N.A. | N.A. | 1.41 [1.20-1.66] | N.A. | N.A. | 1.61 [1.36-1.90] | N.A. | N.A. | 1.48 [1.26-1.74] |
| Adjusted case-case-time-control[a] | N.A. | N.A. | 1.37 [1.16-1.61] | N.A. | N.A. | 1.57 [1.32-1.86] | N.A. | N.A. | 1.45 [1.23-1.70] |

**Abbreviations:** CI, confidence interval; CKD, chronic kidney disease; HFRS, Hospital Frailty Risk Score. [a]Adjusted for time-vary exposure to antidepressants, antipsychotics, benzodiazepines and opioids.

© 2024 Leung MTY et al. *JAMA Network Open*.
