# Supplementary material for: Gabapentinoids and Risk of Hip Fracture
Source: JAMA Netw Open. 2024 Nov 13;7(11):e2444488. doi: 10.1001/jamanetworkopen.2024.44488 (PMC11561685; doi:10.1001/jamanetworkopen.2024.44488)
Supplement: Supplement 2. — Data Sharing Statement [file jamanetwopen-e2444488-s002.pdf]

Data Sharing Statement

Leung. Gabapentinoids and Risk of Hip Fracture. *JAMA Netw Open*. Published November 13, 2024. doi:10.1001/jamanetworkopen.2024.44488

Data
Data available: No
